# Supplementary material for: Response of the competitive balance model to the external field
Source: PLoS One. 2023 Aug 4;18(8):e0289543. doi: 10.1371/journal.pone.0289543 (PMC10403139; doi:10.1371/journal.pone.0289543)
Supplement: S1 Appendix — Part A contains the mathematical calculations and explicit form for the two-body interactions (q). Part B elaborates on the detailed mathematical computations for the Mean-Field solution of three-body interactions (〈E△〉). (PDF) [file pone.0289543.s001.pdf]

Appendix

A Mean-Field solution for two-body term q

At first step, we separate all terms that contain σ_{jk} or σ_{ki}:

$$\mathcal{H} = \mathcal{H}_{\wedge_{jki}} + \mathcal{H}'' \tag{1}$$

$$\begin{aligned}
-\mathcal{H}_{\wedge_{jki}} &= Re\left[\sigma_{jk}\sum_{\ell\neq i,j,k}\sigma_{j\ell}\sigma_{\ell k}\right] + Im\left[\sigma_{jk}\sum_{\ell\neq i,j,k}\sigma_{j\ell}\sigma_{\ell k}\right] + Re\left[\sigma_{ki}\sum_{\ell\neq i,j,k}\sigma_{k\ell}\sigma_{\ell i}\right] \\
&\quad + Im\left[\sigma_{ki}\sum_{\ell\neq i,j,k}\sigma_{k\ell}\sigma_{\ell i}\right] + Re\left(\sigma_{ij}\sigma_{jk}\sigma_{ki}\right) + Im\left(\sigma_{ij}\sigma_{jk}\sigma_{ki}\right) + h(\sigma_{jk}^2 + \sigma_{ki}^2) \qquad (2) \\
&\overset{MF}{\approx} Re\left[\sigma_{jk}(N-3)q\right] + Im\left[\sigma_{jk}(N-3)q\right] + Re\left[\sigma_{ki}(N-3)q\right] + Im\left[\sigma_{ki}(N-3)q\right] \\
&\quad + Re\left(\sigma_{ij}\sigma_{jk}\sigma_{ki}\right) + Im\left(\sigma_{ij}\sigma_{jk}\sigma_{ki}\right) + h(\sigma_{jk}^2 + \sigma_{ki}^2)
\end{aligned}$$

Different modes that these two links can take are

$$\begin{aligned}
&-\mathcal{H}_{\wedge_{ikj}}(\sigma_{jk} = +1, \sigma_{ki} = +1) = 2(N-3)(q_r + q_i) + p_r + p_i + 2h \\
&-\mathcal{H}_{\wedge_{ikj}}(\sigma_{jk} = -1, \sigma_{ki} = -1) = -2(N-3)(q_r + q_i) + p_r + p_i + 2h \\
&-\mathcal{H}_{\wedge_{ikj}}(\sigma_{jk} = +1, \sigma_{ki} = -1) = -(p_r + p_i) + 2h \;\rightarrow\; (\times 2) \\
&-\mathcal{H}_{\wedge_{ikj}}(\sigma_{jk} = +i, \sigma_{ki} = +i) = 2(N-3)(q_r - q_i) - (p_r + p_i) - 2h \\
&-\mathcal{H}_{\wedge_{ikj}}(\sigma_{jk} = -i, \sigma_{ki} = -i) = 2(N-3)(-q_r + q_i) - (p_r + p_i) - 2h \\
&-\mathcal{H}_{\wedge_{ikj}}(\sigma_{jk} = +i, \sigma_{ki} = -i) = p_r + p_i - 2h \;\rightarrow\; (\times 2) \\
&-\mathcal{H}_{\wedge_{ikj}}(\sigma_{jk} = +1, \sigma_{ki} = +i) = 2(N-3)q_r + p_r - p_i \;\rightarrow\; (\times 2) \\
&-\mathcal{H}_{\wedge_{ikj}}(\sigma_{jk} = +1, \sigma_{ki} = -i) = 2(N-3)q_i - p_r + p_i \;\rightarrow\; (\times 2) \\
&-\mathcal{H}_{\wedge_{ikj}}(\sigma_{jk} = -1, \sigma_{ki} = +i) = -2(N-3)q_i - p_r + p_i \;\rightarrow\; (\times 2) \\
&-\mathcal{H}_{\wedge_{ikj}}(\sigma_{jk} = -1, \sigma_{ki} = -i) = -2(N-3)q_r + p_r - p_i \;\rightarrow\; (\times 2)
\end{aligned} \tag{3}$$

By substituting above relations into Eq. 15, we abtain following equation:

$$\langle \sigma_{jk}\sigma_{ki} \rangle \overset{MF}{\approx} \frac{F(p,q;N,\beta,h)}{G(p,q;N,\beta,h)}, \tag{4}$$

where

$$\begin{aligned}
F(p,q;N,\beta,h) &= e^{\beta[2(N-3)(q_r+q_i)+p_r+p_i+2h]} + e^{\beta[-2(N-3)(q_r+q_i)+p_r+p_i+2h]} - 2e^{\beta[-(p_r+p_i)+2h]} \\
&\quad - e^{\beta[2(N-3)(q_r-q_i)-(p_r+p_i)-2h]} - e^{\beta[2(N-3)(-q_r+q_i)-(p_r+p_i)-2h]} + 2e^{\beta[p_r+p_i-2h]} \\
&\quad + 2i\,e^{\beta[2(N-3)q_r+p_r-p_i]} - 2i\,e^{\beta[2(N-3)q_i-p_r+p_i]} - 2i\,e^{\beta[-2(N-3)q_i-p_r+p_i]} \\
&\quad + 2i\,e^{\beta[-2(N-3)q_r+p_r-p_i]},
\end{aligned} \tag{5}$$

$$\begin{aligned}
G(p,q;N,\beta,h) &= e^{\beta[2(N-3)(q_r+q_i)+p_r+p_i+2h]} + e^{\beta[-2(N-3)(q_r+q_i)+p_r+p_i+2h]} + 2e^{\beta[-(p_r+p_i)+2h]} \\
&\quad + e^{\beta[2(N-3)(q_r-q_i)-(p_r+p_i)-2h]} + e^{\beta[2(N-3)(-q_r+q_i)-(p_r+p_i)-2h]} + 2e^{\beta[p_r+p_i-2h]} \\
&\quad + 2e^{\beta[2(N-3)q_r+p_r-p_i]} + 2e^{\beta[2(N-3)q_i-p_r+p_i]} + 2e^{\beta[-2(N-3)q_i-p_r+p_i]} \\
&\quad + 2e^{\beta[-2(N-3)q_r+p_r-p_i]}.
\end{aligned} \tag{6}$$

B Mean-Field solution for three body interactions

Similar to the previous sections, at first, we must separate the sentences that contain links σ_{ij}, σ_{jk}, σ_{ki}:

$$\mathcal{H} = \mathcal{H}_{\triangle_{ijk}} + \mathcal{H}''' \tag{7}$$

$$\begin{aligned} r \equiv \langle \sigma_{ij}\sigma_{jk}\sigma_{ki} \rangle &= \frac{1}{Z}\sum_{G} \sigma_{ij}\sigma_{jk}\sigma_{ki} e^{-\beta\mathcal{H}(G)} \\ &= \frac{\sum_{\{\sigma\neq\sigma_{ij},\sigma_{jk},\sigma_{ki}\}} e^{-\beta\mathcal{H}'''} \sum_{\{\sigma_{ij},\sigma_{jk},\sigma_{ki}=\pm1,\pm i\}} \sigma_{ij}\sigma_{jk}\sigma_{ki} e^{-\beta\mathcal{H}_{\triangle_{ikj}}}}{\sum_{\{\sigma\neq\sigma_{ij},\sigma_{jk},\sigma_{ki}\}} e^{-\beta\mathcal{H}'''} \sum_{\{\sigma_{ij},\sigma_{jk},\sigma_{ki}=\pm1,\pm i\}} e^{-\beta\mathcal{H}_{\triangle_{ikj}}}} \end{aligned} \tag{8}$$

$$\begin{aligned} -\mathcal{H}_{\triangle_{ijk}} = {} & Re\left[\sigma_{ij}\sum_{\ell\neq i,j,k}\sigma_{i\ell}\sigma_{\ell j}\right] + Im\left[\sigma_{ij}\sum_{\ell\neq i,j,k}\sigma_{i\ell}\sigma_{\ell j}\right] + Re\left[\sigma_{jk}\sum_{\ell\neq i,j,k}\sigma_{j\ell}\sigma_{\ell k}\right] \\ & + Im\left[\sigma_{jk}\sum_{\ell\neq i,j,k}\sigma_{j\ell}\sigma_{\ell k}\right] + Re\left[\sigma_{ki}\sum_{\ell\neq i,j,k}\sigma_{k\ell}\sigma_{\ell i}\right] + Im\left[\sigma_{ki}\sum_{\ell\neq i,j,k}\sigma_{k\ell}\sigma_{\ell i}\right] \\ & + Re\,(\sigma_{ij}\sigma_{jk}\sigma_{ki}) + Im\,(\sigma_{ij}\sigma_{jk}\sigma_{ki}) + h(\sigma_{ij}^2 + \sigma_{jk}^2 + \sigma_{ki}^2) \\ \overset{MF}{\approx} {} & Re\,[\sigma_{ij}(N-3)q] + Im\,[\sigma_{ij}(N-3)q] + Re\,[\sigma_{jk}(N-3)q] + Im\,[\sigma_{jk}(N-3)q] \\ & + Re\,[\sigma_{ki}(N-3)q] + Im\,[\sigma_{ki}(N-3)q] + Re\,(\sigma_{ij}\sigma_{jk}\sigma_{ki}) + Im\,(\sigma_{ij}\sigma_{jk}\sigma_{ki}) \\ & + h(\sigma_{ij}^2 + \sigma_{jk}^2 + \sigma_{ki}^2) \end{aligned} \tag{9}$$

$$r_r = Re(r), \qquad r_i = Im(r), \tag{10}$$

$$\langle E_\triangle \rangle = r_r + r_i. \tag{11}$$

So we have:

$$\langle E_\triangle \rangle \overset{MF}{\approx} \frac{V(q;N,\beta,h)}{W(q;N,\beta,h)}, \tag{12}$$

where:

$$\begin{aligned} V(q;N,\beta,h) = {} & -3e^{\beta[-(N-3)(3q_r-q_i)+1-h]} - e^{\beta[-3(N-3)(q_r-q_i)+1-3h]} - 3e^{\beta[-(N-3)(q_r+3q_i)+1+h]} \\ & - 3e^{\beta[-(N-3)(q_r+q_i)+1+3h]} - 6e^{\beta[-(N-3)(q_r-q_i)+1+h]} - 3e^{\beta[(N-3)(q_r-3q_i)+1-h]} \\ & + 3e^{-\beta[(N-3)(q_r-3q_i)+1+h]} - 3e^{\beta[(N-3)(q_r-q_i)+1-3h]} - 6e^{\beta[(N-3)(q_r+q_i)+1-h]} \\ & + 6e^{-\beta[(N-3)(q_r+q_i)+1+h]} - 3e^{\beta[(N-3)(3q_r+q_i)+1+h]} - e^{\beta[3(N-3)(q_r+q_i)+1+3h]} \\ & + 6e^{\beta[(N-3)(q_r-q_i)-1+h]} + 3e^{\beta[(N-3)(q_r+q_i)-1+3h]} + 3e^{-\beta[(N-3)(q_r+3q_i)-1+h]} \\ & + e^{\beta[3(N-3)(q_r-q_i)-1-3h]} + 3e^{\beta[-(N-3)(3q_r+q_i)-1+h]} + 3e^{-\beta[-(N-3)(3q_r-q_i)+1+h]} \\ & + e^{\beta[-3(N-3)(q_r+q_i)-1+3h]} + 3e^{-\beta[-(N-3)(q_r-q_i)-1-3h]} \end{aligned} \tag{13}$$

$$\begin{aligned} W(q;N,\beta,h) = {} & 3e^{\beta[-(N-3)(3q_r-q_i)+1-h]} + e^{\beta[-3(N-3)(q_r-q_i)+1-3h]} + 3e^{\beta[-(N-3)(q_r+3q_i)+1+h]} \\ & + 3e^{\beta[-(N-3)(q_r+q_i)+1+3h]} + 6e^{\beta[-(N-3)(q_r-q_i)+1+h]} + 3e^{\beta[(N-3)(q_r-3q_i)+1-h]} \\ & + 3e^{-\beta[(N-3)(q_r-3q_i)+1+h]} + 3e^{\beta[(N-3)(q_r-q_i)+1-3h]} + 6e^{\beta[(N-3)(q_r+q_i)+1-h]} \\ & + 6e^{-\beta[(N-3)(q_r+q_i)+1+h]} + 3e^{\beta[(N-3)(3q_r+q_i)+1+h]} + e^{\beta[3(N-3)(q_r+q_i)+1+3h]} \\ & + 6e^{\beta[(N-3)(q_r-q_i)-1+h]} + 3e^{\beta[(N-3)(q_r+q_i)-1+3h]} + 3e^{-\beta[(N-3)(q_r+3q_i)-1+h]} \\ & + e^{\beta[3(N-3)(q_r-q_i)-1-3h]} + 3e^{\beta[-(N-3)(3q_r+q_i)-1+h]} + 3e^{-\beta[-(N-3)(3q_r-q_i)+1+h]} \\ & + e^{\beta[-3(N-3)(q_r+q_i)-1+3h]} + 3e^{-\beta[-(N-3)(q_r-q_i)-1-3h]} \end{aligned} \tag{14}$$
